# Proof-of-concept of a laser mounted endoscope for touch-less navigated procedures

**DOI:** 10.1002/lsm.22148

**Published:** 2013-06-04

**Authors:** Florian Kral, Oezguer Gueler, Martina Perwoeg, Zoltan Bardosi, Elisabeth J Puschban, Herbert Riechelmann, Wolfgang Freysinger

**Affiliations:** Department of Otorhinolaryngology, Medical University InnsbruckInnsbruck, Austria

**Keywords:** computer assisted surgery, image processing, minimal invasive surgery, neurosurgery, sinus surgery

## Abstract

**Background and Objectives** During navigated procedures a tracked pointing device is used to define target structures in the patient to visualize its position in a registered radiologic data set. When working with endoscopes in minimal invasive procedures, the target region is often difficult to reach and changing instruments is disturbing in a challenging, crucial moment of the procedure. We developed a device for touch less navigation during navigated endoscopic procedures.

**Materials and Methods** A laser beam is delivered to the tip of a tracked endoscope angled to its axis. Thereby the position of the laser spot in the video-endoscopic images changes according to the distance between the tip of the endoscope and the target structure. A mathematical function is defined by a calibration process and is used to calculate the distance between the tip of the endoscope and the target. The tracked tip of the endoscope and the calculated distance is used to visualize the laser spot in the registered radiologic data set.

**Results** In comparison to the tracked instrument, the touch less target definition with the laser spot yielded in an over and above error of 0.12 mm. The overall application error in this experimental setup with a plastic head was 0.61 ± 0.97 mm (95% CI −1.3 to +2.5 mm).

**Conclusion** Integrating a laser in an endoscope and then calculating the distance to a target structure by image processing of the video endoscopic images is accurate. This technology eliminates the need for tracked probes intraoperatively and therefore allows navigation to be integrated seamlessly in clinical routine. However, it is an additional chain link in the sequence of computer-assisted surgery thus influencing the application error. Lasers Surg. Med. 45:377–382, 2013. © 2013 Wiley Periodicals, Inc.

## INTRODUCTION

Computer assisted surgery (CAS) is used to provide surgeons with positional information of a tracked tool in radiologic imagery [1]. After data acquisition the image stack is registered to the patient [2, 3] and navigation tools such as pointers or registered instruments are tracked by optical or electromagnetic tracking devices [4]. This technology is used routinely in many disciplines for minimal invasive approaches and often combined with video-endoscopes for keyhole procedures in neurosurgery [5,6], otorhinolaryngology [7–9], orthopedics [10,11] or pulmology [12,13]. During endoscopic interventions the surgeon has to change from surgical instruments to tracked tools for positional information in the radiologic data. When working with angled or flexible endoscopes, frequently there is no pointer available to reach the region of interest. For example in navigated transbronchial procedures a tracked probe can be inserted through the endoscope’s working channel [14]. Some navigation systems allow the users to define a distance from the tip of the tracked endoscope. The user-defined distance is then added to the tip of the endoscope in its axis and the resulting “virtual” position is visualized in the radiologic images, which is called “virtual tip” [15]. In any case, the user has to change instruments or software settings thus interrupting the standard workflow of surgical procedures.

This study was performed to proof the concept of a laser-mounted endoscope for touch-less image guided endoscopic procedures. In detail we questioned, with which accuracy the distance of a computer navigated endoscope from an inner body surface could be measured employing the position of a laser spot from the image of a video-endoscopic image. The advantage of this technology is that the need to change to a pointing tracked instrument is not necessary any more. Thus, navigation is seamlessly integrated into surgery.

## MATERIALS AND METHODS

The experiments described were performed with a plastic skull (Somso QS 1, Somso, Coburg, Germany) and therefore did not require clearance by the local ethics committee.

### Modified Endoscope (Naviscope)

**Figure 1:**
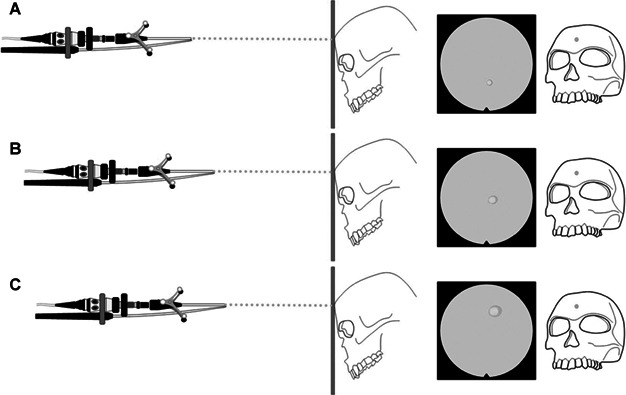
Working principle of laser mounted endoscope: The laser spot changes its position within the endoscopic image when is moved towards or away from a target structure. The laser beam is in a predefined angle to the optical axis of the endoscope. The center of the laser spot is determined by image processing and after calibration the distance from the tip of the endoscope to the target structure can be calculated.

A zero degree video-endoscope (Wolf, Tuttlingen, Germany) was used for modifications. A metal tube with 1 mm diameter was spot-welded to the tip of the endoscope’s shaft (Wolf). Figure 1 shows the light pipe connecting the tip of the endoscope with the laser pointer (Techlasers, Hong Kong) via a coupler mounted directly onto the camera head (R. Wolf 5520.201, Wolf). A green laser pointer provided an average stable output power of 5 mW, a wavelength of 532 nm, a beam divergence <1.2 m Radiant at aperture and a beam diameter <1.2 mm. The laser beam is emitted from the metal tube angled to the endoscope’s optical axis. The whole construction (endoscope with shaft, metal tube, laser pointer and tracker) was fixed in an endoscope holder (Wolf), which was mounted on the operating table (Brumaba, Wolfratshausen, Germany). When activated, the laser spot is seen in the video-endoscopic image. When moving the endoscope towards to or away from an object, the laser spot changes its position within the video-endoscopic image (Fig. 1). The endoscopic image from the digital video system was integrated in the navigation system using the S-VHS standard and PAL system.

### Imaging

Prior to the radiologic imaging, ten titanium screws were implanted evenly distributed in a plastic skull to serve as fiducials for registration and alternately as targets for measurements. A CT scan of the plastic skull was performed with a Siemens Somatom 4 row CT (Siemens, Erlangen, Germany) with 1 mm slice thickness (140 kV, 220 mAs). The image data was transferred to the navigation system via Ethernet.

### Navigation

**Figure 2:**
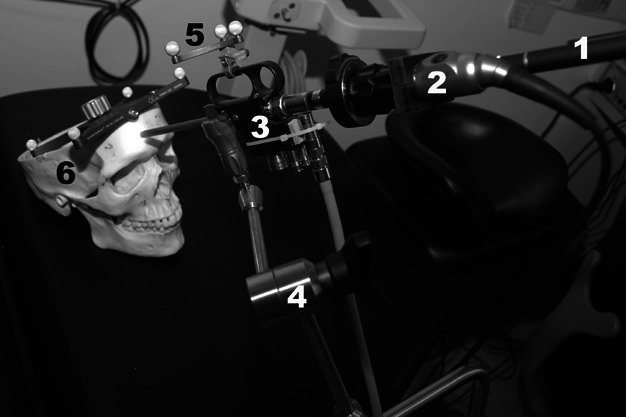
Setup: The laser pointer (1) is mounted firmly to the digital video camera (2) and the laser beam is delivered to the tip of the endoscope (3) that is placed in an endoscope holder (4). For localization a tracker (5) is fixed to the endoscope and a dynamic reference frame for “patient tracking” is attached to the plastic skull (6).

We used a Medtronic S7 navigation system (Medtronic, Minneapolis, MN) with passive optical tracking. The laser mounted endoscope was tracked using the Suretrak® system (Medtronic). The Suretrak® system consists of star like clamps, which can be fixed to surgical instruments. Passive spheres are attached to the clamps and by calibrating the instrument in the navigation software, the tip of the instrument, that is the laser mounted endoscope, can be tracked and visualized in the radiologic data. The image data was registered by pair-point matching using the prior implanted titanium screws in the plastic skull. For tracking, a dynamic reference frame was fixed to the plastic skull and positioned on the operating table (Fig. 2). Direct line of sight was established between the tracking components of the navigation system.

### Image Processing

The video-endoscopic images were transferred via S-VHS to the navigation system and simultaneously streamed via the Fire Wire interface to a standard PC system (Intel Core2Duo CPUe6550; 2.3 GHz; 4 GB RAM; 150 HDD; Fedora 10). From the video a still image was generated on the PC (VLC media player, www.videolan.org/vlc) and stored. A Matlab script was developed (Matlab 7.6, R2008a, Mathworks Inc., MA) for image post-processing to determine the horizontal position of the laser spot’s center pixel within the endoscopic image.

### Working Principle and Measurements

When moving the endoscope towards to or away from the surface of the plastic skull the position of the laser spot changes within the video-endoscopic image, see Figure 1. The position of the laser spot in the video-endoscopic image was used to determine the distance of the endoscope to the target surface. When tracking the tip of the endoscope and adding the calculated distance of the laser spot from the tip of the endoscope along the endoscope axis, the spatial position of the laser spot can be visualized in the multiplanar view of the registered image data by the navigation system.

Prior to the measurements the setup was calibrated. To calibrate the system, the laser-mounted endoscope was fixated at five different distances from the skull’s surface and the video-endoscopic images were stored respectively. The distances defined by the virtual tip were correlated to the pixel position of the laser spot’s center, which resulted in a lookup table. A mathematical approximation function was calculated on base of the pixel position and the according known distance and served as calculation formula for the following measurements. Since the lens distortion is non-linear, the data from the lookup table were approximated using a second-degree polynomial function:

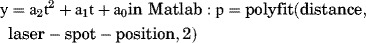


**Figure 3:**
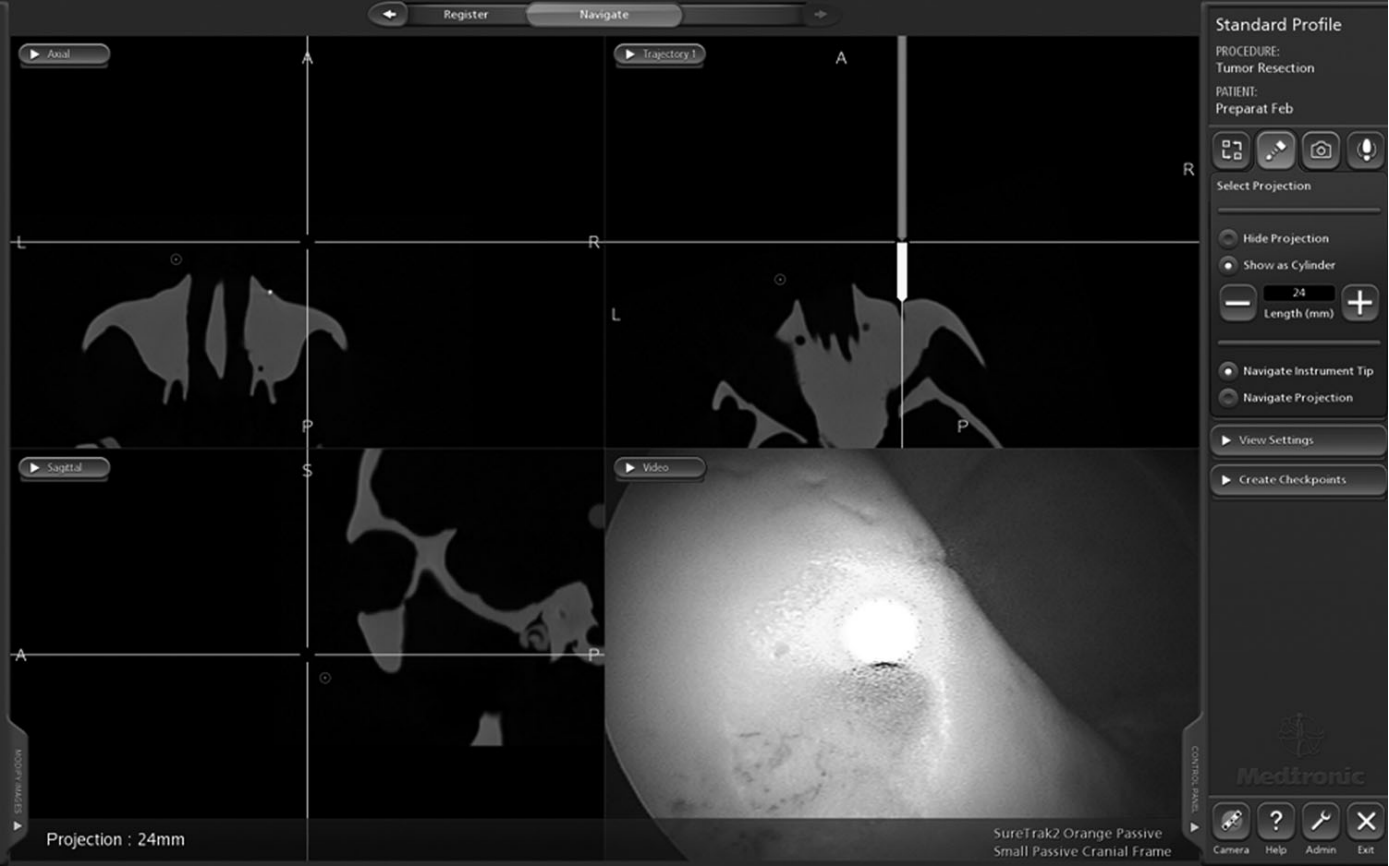
A screenshot of the navigation system is shown. The laser spot was placed directly beside the infraorbital foramen in the endoscopic view. The center of the laser spot is determined (black dot) and the distance from the tip of the endoscope to the surface of the plastic skull was calculated. The computed distance of 24 mm was confirmed by the “virtual tip” application of the navigation system and is shown in a trajectory view.

For measurements the endoscope was placed at an unknown distance from the surface of the plastic skull and fixed with the endoscope holder. To reduce user related errors, the endoscope was fixed in an endoscope holder and was used in parallel as tracked instrument for measuring the distance with the navigation system’s measurement tool (“virtual tip”). The endoscopic image with the laser spot was frozen and the distance from the tip of the endoscope to the surface of the plastic skull was calculated in Matlab. Simultaneously the distance was determined using the “virtual tip” application. This application allows to virtually prolonging the instrument (i.e., the video-endoscope) along its axis in millimeter steps (Fig. 3). Both distances and the difference were recorded. The calibration of each setup with 10 distances and the subsequent measurements of ten unknown distances were repeated ten times. Distance measurements were independent from calibration measurements and performed at different distances. Exact conformance resulted in a deviation (error) of 0 mm, if the device measured a shorter distance than the actual distance, negative values resulted, otherwise positive values.

### Statistics

All data are presented in millimeter. Data distribution was visually checked employing histograms. For each of the 10 distances, calibrations and measurements were performed in 10 repeats. Outcome variables were (a) the raw distances measured by the Naviscope and (b) measurement errors, that is differences of the actual distance measured with the virtual tip and the distances measured by the Naviscope. As a measure of consistency, intraclass correlation coefficient of 10 repeats of the Naviscope distance measurements was calculated in a two-way mixed model treating actual distances as random (Shrout and Fleiss convention ICC(3,1)). For further data analysis, the repeats of the Naviscope distances and measurement errors were averaged for each of the 10 distances. Means and standard deviations of the averaged measurements were then calculated.

## RESULTS

For each distance, 10 repeats of calibrations and Naviscope measurements were performed. Graphical representation revealed a symmetric bell curve shaped curve suggesting normal distribution. Over all distances together, Naviscope measurements had an ICC of almost 1 (0.999, 95% CI 0.997–1,000) suggesting a high consistency of Naviscope measurements. The ICC slightly decreased (worsened) with increasing distances of the Naviscope from the target.

Over all distances, the mean Naviscope measurement error was 0.61 ± −0.97 mm (95% CI −1.3 to +2.5 mm).

Exploiting the difference between the calculated distance from the image processing of the video-endoscopic image and the measured distance with the probe and the virtual tip, the overall accuracy was 0.12 mm. The standard deviation (SD) was ±0.32 mm and the confidence interval (CI) was 0.62 mm. In eight of ten measurements all differences were within the range of ±1 mm. The accuracy of the calculated distances was not normal distributed. Except from the measurements in distance of 40 mm, all measurements were within the range of ±2 mm.

**Figure 4:**
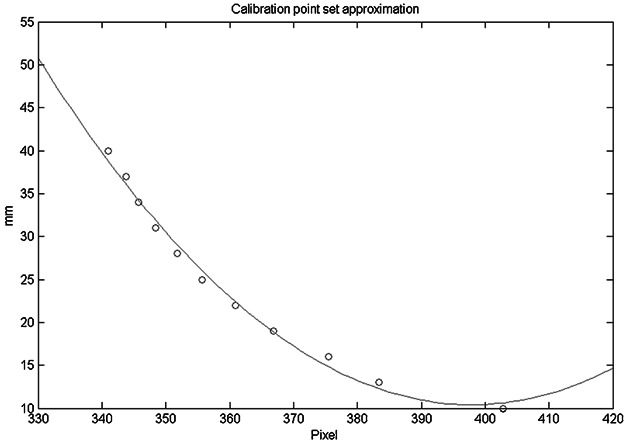
The calibration curve of the Naviscope in the endoscopic image is shown, where the optical center is pixel position 360. The small circles result from the position of the laser spot’s center pixel (*x*-axis) according to the distance (*y*-axis). During calibration a mathematical approximation is performed and shown in this figure. The mathematical function is used to calculate the distance from the tip of the endoscope to the target structure to visualize its position in the medical imagery. The curve shows, that the laser spot crosses the optical center of the endoscope. The angle of the laser was chosen for a symmetric position around the optical center of the laser spots at distances between 10 and 40 mm. Pixel positions outside the predefined working area (10–40 mm) were not considered for calibration and measurements.

Exemplarily, one polynomial function of a look-up table, derived from the calibration process, is given: 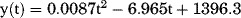 (Fig. 4).

Since the applied polynomial fitting finds the coefficients in a least square sense, the root mean square (RMS) value is an appropriate quality measure. For the above example the RMS value was 0.84 mm.

## DISCUSSION

The application error in this setup was comparable with optically tracked pointing devices [16,17] and is understood as results only achievable under an experimental setup with a plastic head. The application error under real conditions will vary depending on the known error sources of navigation such as imaging, registration, user and tracking. These error sources in mind, this setup was chosen to overcome these limitations, as the preconditions were identical for measurements with the tracked endoscope and the laser spot. The overall accuracy of the distance measuring with the laser spot by image processing (0.12 mm) is an additional error to the overall application error, however in return without the need of a pointing device. Instead of defining the laser spot with triangulation, which was used earlier in sighting devices of rifles and by other authors [18], image processing can be employed even to angled endoscopes. The advantage of projecting a single point is the simple technology and the potential to navigate to point-like anatomical structures *in vivo* and in the patient’s data set. This error needs to be compared to the error inherent to a navigated probe, which is typically calibrated with ∼0.3 mm and may, furthermore, be subject to significant bending forces while measuring. In view of this, the Naviscope does bring a significant improvement of application accuracy.

The target registration error (TRE) [19] is not affected by the fact which type of navigated probe is used; it is dominated exclusively by the geometrical configuration of the fiducial markers and the Fiducial Localization Error, FLE [19].

The aim of this device is to delete the pointer without replacement from navigated endoscopic procedures for a better intraoperative usability, but the distance calculation using a laser is a possible additional source of error to the overall application error. The laser measurement error is however independent from other sources of error affecting the TRE such as tracking, registration, imaging and user. Therefore a direct comparison of distance measurements from the tip of the endoscope to the target structures using the virtual tip functionality and the laser distance measurements was chosen.

The optical characteristics of the endoscope lens are not sufficiently characterized to calculate the distance between endoscope tip and the target structure. An accurate mathematical function of the optics would certainly allow us to eliminate the calibration step. We work on algorithms to characterize the video-endoscopic image to further improve the accuracy. For this reason, the system is calibrated using look-up tables and an approximation with a second-degree order polynomial. From basic geometrical considerations it is clear that the calibration curve will be symmetric over the center of the image as the endoscope distortion is radially symmetric [20].The shape of the data points suggested using a second order polynomial; this will avoid over fitting the data, as the fine modulations in the calibration curve are most likely due to noise in the measurements.

The virtual tip was used to create the look up table instead of example a coordinate measuring machine, because all navigation errors like registration, imaging and tracking are identical for measuring the distance with the virtual tip but also when calculating the distance in unknown positions from the endoscopic images in Matlab. An important limitation of the study is that irregular surfaces may result in distorted spot appearances and reduced accuracy. If the laser spot falls on an irregular anatomical surface, the algorithm is designed to extract the center of gravity of the reflection. Thus by design the anatomic structure that reflects most of the light is shown in the navigated patient images. If an illuminated object lies in the line of sight it is either of surgical interest or it obstructs the view to the surgical site. In the first case, navigation will work sufficiently, in the latter the object will have to be resected; alternatively the viewing position of the endoscope will have to be changed. In summary, the advantage of the Naviscope technology is that it will work on all surfaces (irregular or planar) that sufficiently reflect green laser light. Green laser light gives a nice bright spot on fresh tissue and even in blood. If the laser shines on a “pool of blood,” this technology would track the laser light reflection onto the surface of the pool. The ground of the pool is inaccessible to this technology as for human vision. If one intended to develop a technology unobstructed by blood, a different part of the optic spectrum should be used, to which water and hemoglobin are transparent.

This technique is developed for a seamless integration of navigation in endoscopic procedures. A touch-less definition of the target structure by a laser spot can be used in many disciplines (paranasal sinus surgery, skull base surgery, neurosurgical procedures, and bronchoscopy) in particular when the target structure is difficult or hardly reachable with a pointer. As the need for a navigated pointer is abolished and navigation can extend beyond the tip of a tracked endoscope, the Naviscope may offer advantages in neurosurgical procedures such as ventriculoscopy. Moreover, navigated lung biopsies or navigated laparoscopic interventions will benefit from the elimination of tracked probes and the potential to “look behind” the organ walls. The current device is experimental. For a practical solution, the laser will have to be integrated into the illumination fibers of the optics and will have some optics for coupling the light out into the surgical field. The laser light can be brought through the cold-light cable to the endoscope. Alternatively, as we have developed, a sheath to carry the laser fiber can be used. In the first case a complete new design of a rigid endoscope is necessary; in the latter any standard endoscope, to which the sheath fits, can be used. In summary, a more or less standard technological configuration will result.

Combining a navigated endoscope with the proposed laser distance measuring frees surgeons from the need to switch to dedicated tracked probes or surgical instruments. The advantage of this is that the laser spot is always in the endoscopic field of view and so naturally at the focus of the surgeon’s attention. During paranasal sinus surgery with angled scopes it is often difficult to reach the area of interest with a pointer. This novel laser-based navigation technology is superior to standard metal pointers intraoperatively as it implies less interruptions of intraoperative workflow and eliminates dedicated tools exclusively used for pointing at target structures (i.e., the standard metal navigational pointer). Moreover, the laser spot will always be seen and so the difficulties of reaching certain anatomical areas with a mechanical probe are eliminated. During challenging procedures these features are clear benefits for clinicians.
